# Arrhythmogenic cardiomyopathy

**DOI:** 10.1186/s13023-016-0407-1

**Published:** 2016-04-02

**Authors:** Kalliopi Pilichou, Gaetano Thiene, Barbara Bauce, Ilaria Rigato, Elisabetta Lazzarini, Federico Migliore, Martina Perazzolo Marra, Stefania Rizzo, Alessandro Zorzi, Luciano Daliento, Domenico Corrado, Cristina Basso

**Affiliations:** Department of Cardiac, Thoracic and Vascular Sciences, University of Padua, Padua, Italy

## Abstract

Arrhythmogenic cardiomyopathy (AC) is a heart muscle disease clinically characterized by life-threatening ventricular arrhythmias and pathologically by an acquired and progressive dystrophy of the ventricular myocardium with fibro-fatty replacement. Due to an estimated prevalence of 1:2000-1:5000, AC is listed among rare diseases. A familial background consistent with an autosomal-dominant trait of inheritance is present in most of AC patients; recessive variants have also been reported, either or not associated with palmoplantar keratoderma and woolly hair. AC-causing genes mostly encode major components of the cardiac desmosome and up to 50 % of AC probands harbor mutations in one of them. Mutations in non-desmosomal genes have been also described in a minority of AC patients, predisposing to the same or an overlapping disease phenotype. Compound/digenic heterozygosity was identified in up to 25 % of AC-causing desmosomal gene mutation carriers, in part explaining the phenotypic variability. Abnormal trafficking of intercellular proteins to the intercalated discs of cardiomyocytes and Wnt/beta catenin and Hippo signaling pathways have been implicated in disease pathogenesis.

AC is a major cause of sudden death in the young and in athletes. The clinical picture may include a sub-clinical phase; an overt electrical disorder; and right ventricular or biventricular pump failure. Ventricular fibrillation can occur at any stage. Genotype-phenotype correlation studies led to identify biventricular and dominant left ventricular variants, thus supporting the use of the broader term AC.

Since there is no “gold standard” to reach the diagnosis of AC, multiple categories of diagnostic information have been combined and the criteria recently updated, to improve diagnostic sensitivity while maintaining specificity. Among diagnostic tools, contrast enhanced cardiac magnetic resonance is playing a major role in detecting left dominant forms of AC, even preceding morpho-functional abnormalities. The main differential diagnoses are idiopathic right ventricular outflow tract tachycardia, myocarditis, sarcoidosis, dilated cardiomyopathy, right ventricular infarction, congenital heart diseases with right ventricular overload and athlete heart. A positive genetic test in the affected AC proband allows early identification of asymptomatic carriers by cascade genetic screening of family members. Risk stratification remains a major clinical challenge and antiarrhythmic drugs, catheter ablation and implantable cardioverter defibrillator are the currently available therapeutic tools. Sport disqualification is life-saving, since effort is a major trigger not only of electrical instability but also of disease onset and progression. We review the current knowledge of this rare cardiomyopathy, suggesting a flowchart for primary care clinicians and geneticists.

## Arrhythmogenic Cardiomyopathy- key points summary

- AC is a rare (1:2000-1:5000) heredo-familial cardiomyopathy, with an age-related penetrance (usually adolescence-young adulthood)
- Clinical presentation is characterized by ventricular arrhythmias at risk of sudden death. More rarely, right ventricular or biventricular dysfunction leading to heart failure is reported
- Generally referred as right ventricular disease, recognition of left-dominant and biventricular subtypes prompted the use of the broader term AC
- Effort is a trigger of disease onset and progression as well as ventricular arrhythmias
- Disease causing genes mostly encode for desmosomal proteins, although non-desmosomal genes are also described
- The structural substrate of AC consists of progressive myocardial dystrophy with fibro-fatty replacement in the ventricular walls, starting from the subepicardium. It accounts for morpho-functional ventricular wall abnormalities that can be absent in the early stages
- The in vivo demonstration of the structural substrate of AC can be achieved directly by endomyocardial biopsy or indirectly by contrast- enhanced cardiac magnetic resonance (late–enhancement) and electrovoltage anatomic mapping (low voltage area)
- Knowledge of phenocopies that can mimic AC is essential to avoid misdiagnosis
- Shortcomings of 2010 update diagnostic criteria:
  - left ventricular variant is almost missing
  - contrast-enhanced cardiac magnetic resonance and electrovoltage anatomic mapping are not yet part of the diagnostic workup
  - identification of a gene mutation in a patient under evaluation is a major diagnostic criterion (issues of mutation pathogenicity and “genetic load”)
- Life-style modification (avoidance of strenuous effort), anti-arrhythmic drugs, endocardial and epicardial catheter ablation, and implantable cardioverter defibrillator are the usual therapeutic tools
- Risk stratification relies on phenotypic predictors and recommendations for implantable cardioverter defibrillator have been recently provided by a consensus. Prognostic data are not yet available for the left dominant variant
- Abnormal cell-cell adhesion, altered intracellular signaling (Wnt and Hippo pathways) leading to myocyte death and fibro-adipogenesis, gap junction and ion channel remodelling are the pathogenetic theories under investigation
- The identification of a compound known to be an activator of the canonical Wnt signaling pathway by high-throughput drug screening in zebrafish JUP model opens the door for a curative therapy

## Background 

Arrhythmogenic cardiomyopathy (AC) (OMIM #107970; ORPHA247) is a rare disease of the heart muscle characterized by a progressive myocardial dystrophy with fibro-fatty replacement [1–4]. It is a genetically determined cardiomyopathy caused by heterozygous or compound heterozygous mutations in genes mostly encoding proteins of the desmosomal complex (about 50 % of probands). Cases with a recessive trait of inheritance have been reported, either associated or not with skin/hair abnormalities [3, 4]. AC shows an age-related penetrance, manifesting with palpitations, syncope or cardiac arrest usually in adolescence or young adulthood [5] and represents one of the major causes of sudden death (SD) in the young and athlete [1, 6, 7]. High clinical and genetic variability is reported. There is no a single gold standard for the diagnosis, which is mainly based on functional and structural alterations of the right ventricle (RV), fibro-fatty replacement of the myocardium, depolarization and repolarization abnormalities, arrhythmias with the left bundle branch block (LBBB) morphology and family history [8]. Genotype-phenotype correlation studies have recently identified clinical variants characterized by early dominant left ventricular (LV) involvement, besides the classical RV variant, thus supporting the use of the broader term AC [3, 4, 9–11]. In this paper we will review clinical, pathologic and genetic findings of AC, together with diagnosis and treatment. The current knowledge on disease pathogenesis will also be treated.

### Disease names/synonyms

Arrhythmogenic Cardiomyopathy (AC, ACM), Arrhythmogenic RV Cardiomyopathy (ARVC), Arrhythmogenic RV Cardiomyopathy/Dysplasia (ARVC/D), Arrhythmogenic RV Dysplasia (ARVD).

### Definition

More than 20 years elapsed since the 1995 WHO definition and classification of cardiomyopathies [12], the genetic background has been discovered and new diagnostic tools are now available. Thanks to genotype-phenotype correlation, we know that the disease spectrum is wider than previously thought, with LV involvement present and even dominant at early stages. Thus, nowadays the original disease definition could be updated as: “AC is characterized by progressive fibro-fatty replacement of ventricular myocardium, including RV and LV, with relative sparing of the septum. Presentation with arrhythmias and SD is common, particularly in the young. Mutations in genes mostly encoding for desmosomal proteins are found in about half of probands”.

### Epidemiology

The estimated prevalence of AC in the general population ranges from 1:2000 to 1:5000 [3, 5]. Considered in the past an endemic disease in North East Italy (“Venetian disease”), AC is now well recognized in human populations of different ethnicity, but no data are available on its prevalence across various countries. AC affects more frequently males than females (up to 3:1), despite a similar prevalence of carrier status between sexes, and becomes clinically overt most often in the second-fourth decade of life [3, 5]. More rarely, symptoms and signs can appear before puberty or in the elderly. However, occasionally the first clinical manifestations arise even in patients >70, but the diagnosis is often missed because clinicians do not take it into consideration this morbid entity in this older age-group [3].

### Pathological findings

**Fig. 1:**
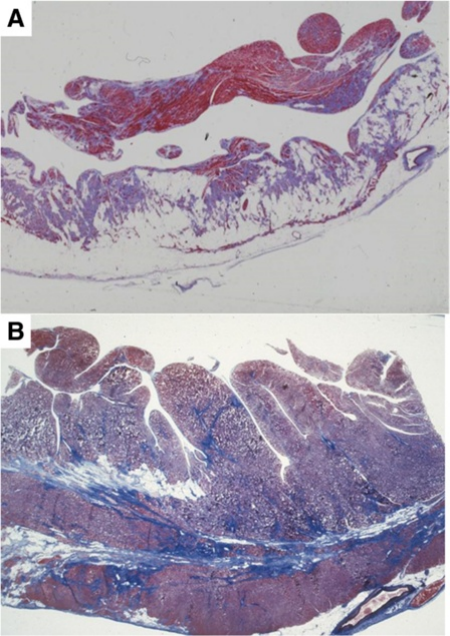
Histopathology of classical RV and left dominant AC variants in AC patients who died suddenly. **a** transmural fibrofatty replacement of the RV free wall **b** mid-mural subepicardial fibro-fatty (mostly fibrous) replacement of the LV postero-lateral free wall

AC is a “structural” cardiomyopathy characterized by dystrophy of the ventricular myocardium with replacement by fibro-fatty tissue [1, 2] (Fig. 1). Myocardial atrophy occurs progressively with time, starts from the epicardium and eventually extends to become transmural. This entity should not be confound with Uhl’s disease, a congenital heart defect in which the RV myocardium fails to develop during embryonic life [9, 13]. The gross pathognomonic features of AC consist of RV aneurysms, whether single or multiple, located in the so-called “triangle of dysplasia” (i.e. inflow, apex and outflow tract) [2, 14]. Nevertheless, grossly normal hearts have been reported in whom only a careful histopathology investigation can reveal AC features. Even cases with isolated or predominant LV involvement [4] are no so rare. Indeed, up to 76 % of the AC hearts studied at post-mortem disclosed a LV involvement, usually limited to the subepicardium or midmural layers of the postero-lateral free wall [2, 15]. Hearts with end-stage disease and congestive heart failure usually show multiple RV aneurysms and huge chamber dilatation, with a higher prevalence of biventricular involvement, while the ventricular septum is mostly spared [2].

Histological examination reveals islands of surviving myocytes, interspersed with fibrous and fatty tissue [1, 2]. Fatty infiltration of the RV is not a sufficient morphologic hallmark of AC [16, 17] and replacement-type fibrosis and myocyte degenerative changes should be always searched for. Myocyte necrosis is seldom evident and may be associated with inflammatory infiltrates [2, 18]. Myocardial inflammation has been reported in up to 75 % of hearts at autopsy. An apoptotic mechanism of myocyte death has been also proven [19, 20]. Rather than being a continuous, ongoing process, disease progression may occur through periodic “acute bursts” of an otherwise stable disease, as to mimic myocarditis or myocardial infarction with normal coronary arteries. In a desmoglein-2 (dsg2) transgenic animal model, myocyte necrosis was demonstrated to be the key initiator of myocardial injury, triggering progressive myocardial damage, followed by an inflammatory response [21]. The detection of viral genomes let to advance an infective viral etiology, but it is most likely that either viruses are innocent bystanders or that myocardial cell degeneration may serve as a milieu favoring viral settlement [22].

### Clinical findings and natural history

In adolescents or young adults, AC usually presents with palpitations, syncope, or cardiac arrest. Then, premature ventricular complexes (PVC) or ventricular tachycardia (VT) with LBBB morphology and T-wave inversion in V_1-_ V_3_ leads on basal electrocardiogram (ECG) are the most common index of suspicion. Less-common presentations are RV or biventricular dilatation, with or without heart failure symptoms, mimicking dilated cardiomyopathy. Clinical manifestations vary with age and stage of disease. Men usually develop a more severe phenotype, most likely because of the effect of vigorous sport activity on disease onset and progression and influence of sex hormones.

Syncope, palpitations and ventricular arrhythmias are the usual presenting symptoms also in the paediatric age [23]. However, non-specific clinical features not infrequently consist of myocarditis or myocardial infarction like-picture with chest pain, dynamic ST-T wave changes on basal 12- lead ECG and myocardial enzymes release with normal coronary arteries [3].

Four phases are recognizable along the natural history of the classic AC variant: 1) “concealed”, with subtle RV structural changes, with or without ventricular arrhythmias; 2) “overt electrical disorder”, with symptomatic life-threatening ventricular arrhythmias associated with clear cut RV morpho-functional abnormalities; 3) “RV failure”, due to progression and extension of RV disease; and 4) “biventricular failure”, caused also by pronounced LV disease [24]. Electrical instability may lead to arrhythmic SD any time during the course of the disease [2, 5, 6, 25–27]. AC has been reported as the second cause of SD in the young and the first cause in competitive athletes in the Veneto Region of Italy [1, 6, 7]. The incidence of SD ranges from 0,08 · to 3,6 % per year in adults with AC [3, 5, 25–27]. While patients with an overt disease phenotype more often experience scar-related re-entrant VT, those with an early stage or a “hot phase” of the disease may present with ventricular fibrillation (VF) due to ongoing myocyte death and reactive inflammation [27]. More recently, gap junction remodelling and sodium channel interference have been advanced in experimental models as alternative substrates for life-threatening arrhythmias even in the pre-phenotypic disease stage [28, 29].

### AC diagnosis

**Fig. 2:**
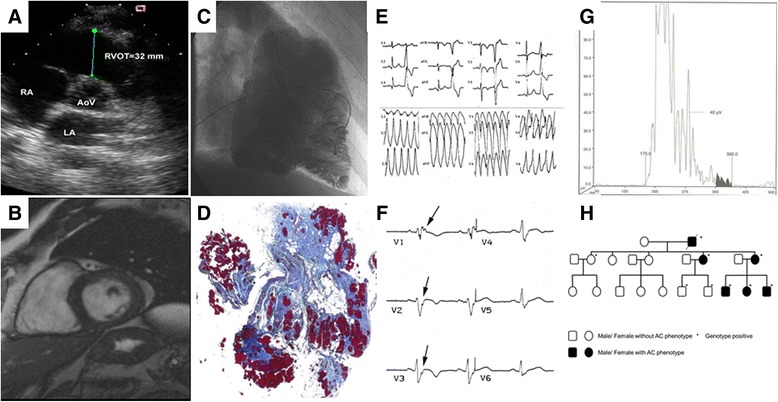
Diagnostic tools to achieve a clinical diagnosis of classical RV AC. **a**, **b**, **c** echocardiography, CMR and angiography showing RV dilatation and aneurysms; **d** tissue characterization through endomyocardial biopsy; **e** 12-lead ECG with inverted T waves V1-V3, LBBB morphology PVCs and VT; **f** post-excitation epsilon wave in precordial leads V1-V3 (arrows); **g** Signal-averaged ECG with late potentials (40 Hz high-pass filtering); **h** Family pedigree with autosomal dominant inheritance of the disease

No single gold-standard is available for AC diagnosis. Multiple criteria are needed, combining different sources of diagnostic information, such as morpho-functional abnormalities (by echocardiography and/or angiography and/or cardiac magnetic resonance-CMR), histopathological features on endomyocardial biopsy (EMB), ECG, arrhythmias and family history, including genetics (Fig. 2). The diagnostic criteria, originally put forward in 1994 [30], have been revised in 2010 to improve diagnostic sensitivity, but with the important prerequisite of maintaining diagnostic specificity (Table 1) [8].

**Table 1:** 2010 Revised Task Force Criteria for AC

I. Global or regional dysfunction and structural alterations*	
Major	
	By 2D echo
	Regional RV akinesia, dyskinesia, or aneurysm
	and 1 of the following (end diastole):
	▪ PLAX RVOT ≥32 mm (corrected for body size [PLAX/BSA] ≥ 19 mm/m2)
	▪ PSAX RVOT ≥36 mm (corrected for body size [PSAX/BSA] ≥21 mm/m2)
	▪ or fractional area change ≤ 33 %
	By CMR
	Regional RV akinesia or dyskinesia or dyssynchronous RV contraction
	and 1 of the following:
	▪ Ratio of RV end-diastolic volume to BSA ≥110 mL/m2 (male) or ≥100 mL/m2 (female)
	▪ or RV ejection fraction ≤40 %
	By RV angiography
	Regional RV akinesia, dyskinesia, or aneurysm
Minor	
	By 2D echo
	Regional RV akinesia or dyskinesia
	and 1 of the following (end diastole):
	▪ PLAX RVOT ≥29 to <32 mm (corrected for body size [PLAX/BSA] ≥16 to <19 m/m2)
	▪ PSAX RVOT ≥32 to <36 mm (corrected for body size [PSAX/BSA] ≥18 to <21 mm/m2)
	▪ or fractional area change >33 % to ≤40 %
	By CMR
	Regional RV akinesia or dyskinesia or dyssynchronous RV contraction
	and 1 of the following:
	▪ Ratio of RV end-diastolic volume to BSA ≥100 to <110 mL/m2 (male) or ≥90 to <100 mL/m2 (female)
	▪ or RV ejection fraction >40 % to ≤45 %
II. Tissue characterization of wall	
Major	
	Residual myocytes <60 % by morphometric analysis (or <50 % if estimated), with fibrous replacement of the RV free wall myocardium in ≥1 sample, with or without fatty replacement of tissue on EMB
Minor	
	Residual myocytes <60 % by morphometric analysis (or <50 % if estimated), with fibrous replacement of the RV free wall myocardium in ≥1 sample, with or without fatty replacement of tissue on EMB
III. Repolarization abnormalities	
Major	
	▪ Inverted T waves in right precordial leads (V1, V2, and V3) or beyond in individuals >14 years of age (in the absence of complete RBBB QRS ≥120 ms)
Minor	
	▪ Inverted T waves in leads V1 and V2 in individuals >14 years of age (in the absence of complete RBBB) or in V4, V5, or V6
	▪ Inverted T waves in leads V1, V2, V3, and V4 in individuals >14 years of age in the presence of complete right RBBB
IV. Depolarization/conduction abnormalities	
Major	
	▪ Epsilon wave (reproducible low-amplitude signals between end of QRS complex to onset of the T wave) in the right precordial leads (V1 to V3)
Minor	
	▪ Late potentials by SAECG in ≥1 of 3 parameters in the absence of a QRS duration of ≥110 ms on the standard ECG
	▪ Filtered QRS duration (fQRS) ≥114 ms
	▪ Duration of terminal QRS <40 μV (low-amplitude signal duration) ≥38 ms
	▪ Root-mean-square voltage of terminal 40 ms ≤20 μV
	▪ Terminal activation duration of QRS ≥55 ms measured from the nadir of the S wave to the end of the QRS, including R’, in V1, V2, or V3, in the absence of complete RBBB
V. Arrhythmias	
Major	
	▪ Nonsustained or sustained VT of LBBB morphology with superior axis (negative or indeterminate QRS in leads II, III, and aVF and positive in lead aVL)
Minor	
	▪ Nonsustained or sustained VT of RVOT configuration, LBBB morphology with inferior axis (positive QRS in leads II, III, and aVF and negative in lead aVL) or of unknown axis
	▪ >500 PVCs per 24 hours (Holter)
VI. Family history	
Major	
	▪ AC confirmed in a first-degree relative who meets current Task Force criteria
	▪ AC confirmed pathologically at autopsy or surgery in a first-degree relative
	▪ Identification of a pathogenic mutation† categorized as associated or probably associated with AC in the patient under evaluation
Minor	
	▪ History of AC in a first-degree relative in whom it is not possible or practical to determine whether the family member meets current Task Force criteria
	▪ Premature SD (35 years of age) due to suspected AC in a first-degree relative
	▪ AC confirmed pathologically or by current Task Force Criteria in second-degree relative

Two major, or one major and two minor, or four minor criteria: definite diagnosis of AC. One major and one minor, or three minor criteria: borderline diagnosis; One major, or two minor criteria from different categories: possible diagnosis

* Hypokinesis is not included in this or subsequent definitions of RV regional wall motion abnormalities for the proposed modified criteria

† A pathogenic mutation is a DNA alteration associated with AC that alters or is expected to alter the encoded protein, is unobserved or rare in a large non-AC control population, and either alters or is predicted to alter the structure or function of the protein or has demonstrated linkage to the disease phenotype in a conclusive pedigree

*Abbreviations*. *BSA*: body surface area; *CMR*: cardiac magnetic resonance; *EMB*: endomyocardial biopsy; *LBBB*: left bundle- branch block; *PLAX*: parasternal long-axis view; *PSAX*: parasternal short-axis view; *PVC*: premature ventricular complex; *RBBB*: right bundle-branch block; *RV*: right ventricle; *RVOT*: RV outflow tract; *SD*: sudden death; *VT*: ventricular tachycardia

Quantitative parameters have been included and abnormalities were defined, based on the comparison with normal subject data. Moreover, T-wave inversion in V_1_-V_3_, and VT with a LBBB morphology with superior or indeterminate QRS axis (either sustained or no sustained), have become major diagnostic criteria; and T-wave inversion in V_1_-V_2_, in the absence of right bundle branch block (RBBB), and in V_1_-V_4_, in the presence of complete RBBB, has been included among the minor criteria. Finally, in the family history category, the confirmation of AC in a first-degree relative, by either meeting current criteria or pathologically (at autopsy or surgery), and the identification of a pathogenic mutation, categorized as associated or probably associated with AC, in the patient under evaluation are considered major criteria. Because of the diagnostic implications, however, caution is highly recommended since the pathogenic significance of a mutation is increasingly questioned (see genetic section) [8].

### AC diagnosis in the pediatric age

AC diagnosis is exceptionally made below the age of 10 [23]. The diagnostic criteria in adults have been demonstrated to be also valid in the pediatric age group, with the exception of inverted T wave on right precordial leads in children < 12 years of age, which may be normal. However, negative results are quite common before adolescent growth is completed, due to absent or limited morpho-functional phenotype, since AC usually shows an age-related penetrance [3, 5]. Follow-up by non-invasive clinical investigation of children who have a family and/or personal history suspicious for AC or healthy gene carriers is recommended on a regular basis, to monitor the pending disease onset in the pubertal period.

### Left dominant AC diagnosis

**Fig. 3:**
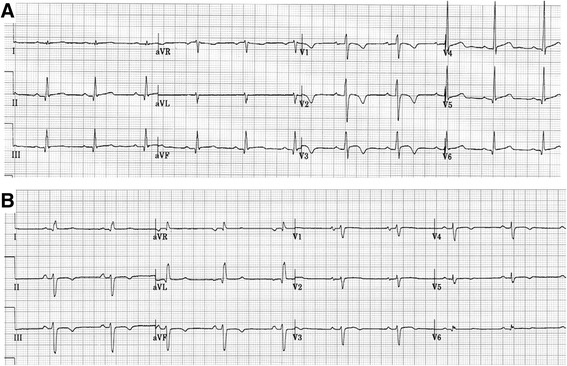
Electrocardiographic features of classical RV vs left dominant AC variants. **a** classical RV AC variant: negative T waves in V1-V3; **b** left dominant AC variant: negative T waves in V4-V6 and inferior leads

**Fig. 4:**
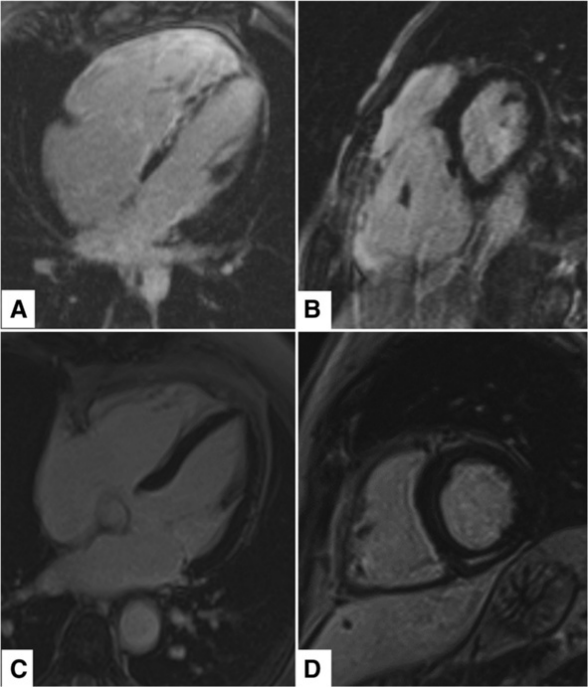
Contrast enhanced CMR of classical RV (**a**, **b**) vs left dominant (**c**, **d**) AC variants. **a**, **b** Four-chamber and short axis views: widespread RV late gadolinium enhancement with septal and LV involvement is notable; **c**, **d** Four chamber and short axis views: late gadolinium enhancement with a mid-mural and sub-epicardial stria is visible in the inferior LV wall

The 2010 criteria acknowledge that RV AC is the best recognized variant of a broad disease spectrum, that includes also LV and biventricular subtypes [8]. While the revised criteria easily catch the classical RV variant, there is lack of specific diagnostic guidelines for non-classical disease patterns, thus explaining the under-recognition of the LV one. The only step forward with respect to the 1994 criteria [30] is that patients with a moderate-to-severe LV dysfunction can now be diagnosed as affected by AC. ECG abnormalities such as lateral or inferolateral T-wave inversion (leads V_5_, V_6_, L_I_, and aVL), low voltage QRS complex on peripheral leads and RBBB/polymorphic ventricular arrhythmias suggest a left-side involvement [31–33] (Fig. 3 and Table 2). Contrast-enhanced CMR is the more sensitive imaging tool to catch LV involvement by non-invasive tissue characterization [33–35]. Late gadolinium enhancement is in fact a far more-sensitive indicator of even early or minor left-sided disease, and is frequently detected in a wall segment without a concomitant morphofunctional abnormality, thus preceding the onset of LV dysfunction or dilatation. Typically, LV late gadolinium enhancement involves the inferolateral and inferoseptal regions, and affects the subepicardial or midwall layers (Fig. 4). Differential diagnosis with dilated cardiomyopathy is mandatory for risk-stratification and familial evaluation purposes. The propensity to electrical instability that exceeds the degree of ventricular dysfunction is typical of left dominant AC, at difference from dilated cardiomyopathy where life-threatening ventricular arrhythmias usually occur in the context of systolic dysfunction with low ejection fraction (<35 %). Moreover, a regional rather than global involvement is more in keeping with AC, particularly when RV abnormalities are also prominent. Thus, a revision of the 2010 Task Force criteria is needed to fill the gap by incorporating features suggesting LV involvement.

**Table 2:** Main clinical features of RVOT tachycardia, RV AC and LV AC

	Idiopathic RVOT tachycardia	RV AC	LV AC
Family history	-	+	+
Cardiocutaneous syndrome	-	+ Naxos syndrome	+ Carvajal syndrome
Desmosomal gene mutations	-	up to 60 %	about 30 %
12 lead ECG	Normal	Normal *or* T-wave inversion V1-V3/V4	Normal *or* T-wave inversion infero-lateral leads
		RBBB varying degree	ε wave II,III, aVF, and/or V4-V6, I, aVL
		S wave delayed upstroke, ε wave V1-V3	Low voltage QRS complexes
SAECG	-	+	-+
Arrhythmia	LBBB morphology PVCs/ventricular arrhythmias with an inferior axis (R wave positive in leads II and III and negative in lead aVL)	LBBB morphology PVCs/ventricular arrhythmias, with inferior, superior and intermediate QRS axis	RBBB morphology PVCs/ventricular arrhythmias
	Single VT morphology, QRS axis inferior	Multiple VT morphologies common	Multiple VT morphologies common
Inducibility at EPS	+-	+	NA
Ventricular volumes	Normal *or* RVOT mild dilatation	Normal *or* mild, moderate or severe RV dilatation ± dysfunction,	Normal *or* mild, moderate or severe LV dilatation ± dysfunction
		RV/LV volume ≥1.2	RV/LV volume <1
Other imaging findings	-	Localized dilatation, WMA, and/or aneurysms in RV	Localized dilatation, WMA, and/or aneurysms in LV
			Non-compacted appearance
EMB	-	+	-+
CMR	-	Fat in RV myocardium	LE in LV myocardium (subepicardial-midmural)
		LE in RV myocardium	
RV Electro-anatomic mapping	-	+	-+
SCD risk	-	+	+

*Abbreviations*. *CMR*: cardiac magnetic resonance; *EMB*: endomyocardial biopsy; *EPS*: electrophysiologic study; *LBBB*: left bundle branch block; *LE*: late enhancement; *LV*: left ventricle; *RBBB*: right bundle branch block; *RV*: right ventricle; *RVOT*: right ventricular outflow tract; *SAECG*: signal averaged ECG; *SCD*: sudden cardiac death; *VT*: ventricular tachycardia; *WMA*: wall motion abnormalities

### Differential diagnosis

**Fig. 5:**
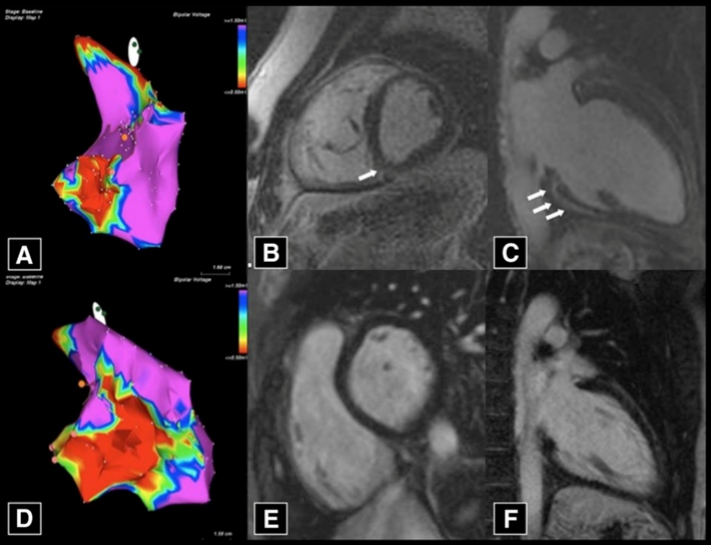
Electroanatomic voltage mapping vs. contrast enhanced CMR in AC. The fibro-fatty scar pathognomonic of AC is indirectly identifiable through either electroanatomic voltage mapping, as low-voltage areas (normal myocardium:purple color), or CMR, as late-enhancement area. While the former is superior in detecting the RV involvement (**a**, **d**), the latter is often the only tool able to detect the frequent LV involvement (**b**, **c**, **e**, **f**) (from Ref 34, with permission)

Common dilemmas in differential diagnosis include myocarditis, sarcoidosis, RV infarction, dilated cardiomyopathy, Chagas disease, Brugada syndrome, pulmonary hypertension and congenital heart disease with right chambers overload [3]. Moreover, one of the main clinical challenge is still differentiation of AC from idiopathic RV outflow tract VT, which is usually benign and non-familial (Table 2) [36]. EMB can be crucial both to rule out the phenocopies such as myocarditis and sarcoidosis, especially when dealing with sporadic forms, and in the setting of negative or doubtful CMR and/or electroanatomic voltage mapping [37]. The latter is an invasive electrophysiological tool that can be performed in selected patients with suspected AC, in the setting of ventricular arrhythmias of RV origin, and/or when contrast-enhanced CMR is negative or doubtful in terms of RV involvement (Fig. 5) [36, 38]. The abnormal low-voltage areas found in AC patients correspond to the loss of electrically active myocardium caused by fibrofatty replacement (“electrical scars”). Noteworthy, because of the wavefront progression of RV fibro-fatty replacement from the epicardium to the endocardium, scar tissue in non-advanced stages may be confined to epicardial/midmural layers, sparing (or reaching focally) the endocardial region. Thus, bipolar endocardial voltage mapping of the RV free wall may underestimate or miss nontransmural low-voltage areas [39].

Finally, in people performing sport activity, AC should be distinguished from so-called “athlete heart”, i.e. physiological adaptation to training with hemodynamic overload. RV enlargement, ECG abnormalities and arrhythmias are well documented in endurance athletes, reflecting the increased hemodynamic load during exercise [3, 40, 41]. Global RV systolic dysfunction and/or regional wall motion abnormalities, such as bulgings or aneurysms, are in keeping more with AC than physiologic ventricular enlargement. The absence of overt structural changes of the RV, frequent PVCs or inverted T waves in precordial leads all support a benign nature.

### AC genes/mutations and diagnostic implications

In 1982, Marcus et al. advocated the inherited nature of AC, with the description of two affected adult cases in the same family [14]. The Padua group then demonstrated that the classical inheritance pattern of AC is autosomal dominant with variable expression and age-dependent penetrance [5, 42]. Inter- and intra-familial phenotype diversity is frequent in AC, with co-existence of both the classic RV and dominant LV pattern in the same family and/or life-threatening ventricular arrhythmias in probands vs a more favorable prognosis in relatives [5, 31, 32]. The first disease-causing gene [43], i.e. the plakoglobin gene (JUP), was identified in a fully penetrant autosomal-recessive form of AC associated with palmoplantar keratosis, also known as Naxos or cardiocutaneous syndrome [44]. Subsequently, mutations of the desmoplakin (DSP) gene were found to cause another autosomal recessive cardiocutaneous syndrome, i.e. Carvajal syndrome [45]. Soon after, heterozygous mutations in the same gene were identified in a dominant form of AC without hair/skin abnormalities [46]. To-date, 11 disease genes have been linked to the AC phenotype, highlighting genetic heterogeneity. Most of mutations in dominant forms have been identified in desmosomal genes including DSP, plakophilin-2 (PKP2), desmoglein-2 (DSG2), desmocollin-2 (DSC2) and JUP [43, 46–50] (Table 3). Only isolated reports showed causal mutations in non-desmosomal genes, such as transmembrane protein 43 (TMEM43), desmin (DES), titin (TTN), Lamin A/C (LMNA), phospholamban (PLN) and αT-catenin (CTNNA3), sometimes with a clinical phenotype similar but not identical to AC, as to be considered phenocopies or overlap syndromes [51–56]. Moreover, mutations in the regulatory region of transforming growth factor beta-3 gene have also been reported [57], but their pathogenicity is still controversial. Finally, ryanodine receptor 2 gene mutations are associated with catecholaminergic polymorphic VT (CPVT-see) rather than with AC, as originally considered [58].

**Table 3:** Genetic background of AC

MIM entry	Locus	Disease gene	Gene	Mode of transmission	Author, year [Reference]	Comment
Desmosomal genes						
#611528	17q21.2	Plakoglobin	JUP	AD/AR	McKoy et al. [43],	AR form: Cardiocutaneous syndrome
#601214						
#607450	6p24.3	Desmoplakin	DSP	AD/AR	Rampazzo et al. [46],	AR form: Cardiocutaneous syndrome
#605676						
#609040	12p11.21	Plakophilin- 2	PKP2	AD/AR	Gerull et al. [47],	
#610193	18q12.1	Desmoglein-2	DSG2	AD/AR	Pilichou et al. [48],	
#610476	18q12.1	Desmocollin-2	DSC2	AD/AR	Syrris et al. [49],	
Non-desmosomal genes						
#600996	1q43	Cardiac Ryanodine Receptor 2	RYR2	AD	Tiso et al. [58],	CPVT (AC phenocopy)
#107970	14q24.3	Transforming growth factor-beta-3	TGFB3	AD	Beffagna et al. [57],	Modifier?
#604400	3p25.1	Transmembrane Protein 43	TMEM43	AD	Merner et al. [51],	
	2q35	Desmin	DES	AD	Van Tintelen et al. [52],	Overlap syndrome (DC and HC phenotype, early conduction disease)
	6q22.31	Phospholamban	PLN	AD	Van der Zwaag et al. [53],	
	2q31.2	Titin	TTN	AD	Taylor et al. [54],	Overlap syndrome (early conduction disease, AF)
	1q22	Lamin A/C	LMNA	AD	Quarta et al. [55],	Overlap syndrome
#615616	10q21.3	alpha-T-catenin	CTNNA3	AD	Van Hengel et al. [56],	

*Abbreviations*. *AD*: autosomal dominant; *AF*: atrial fibrillation; *AR*: autosomal recessive; *CPVT*: catecholaminergic polymorphic ventricular tachycardia; *DC*: dilated cardiomyopathy; *HC*: hypertrophic cardiomyopathy

**Fig. 6:**
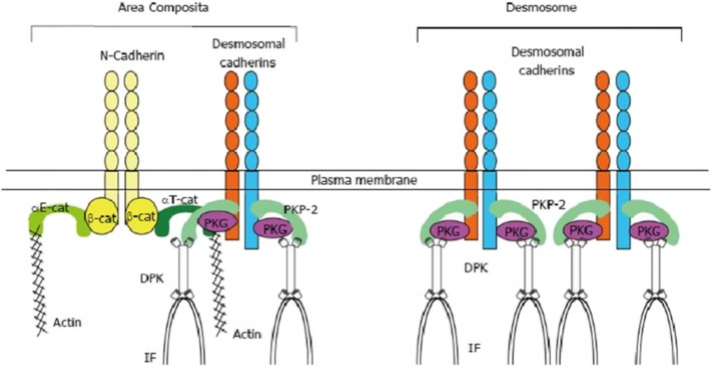
Cadherin-based cell-cell junctions in the cardiomyocytes. Area composita is a mixed-type junctional structure composed of both desmosomal and adherens junctional proteins. Both αE-catenin (αE-cat) and αT-catenin (αT-cat) are present in the area composita at the cardiac intercalated disc, but only αT-catenin was shown to interact directly with PKP2. β-cat: β-catenin; PKG: Plakoglobin; PKP2: Plakophilin-2; DPK: Desmoplakin; IF: Intermediate filaments (From Ref 59, modified)

**Fig. 7:**
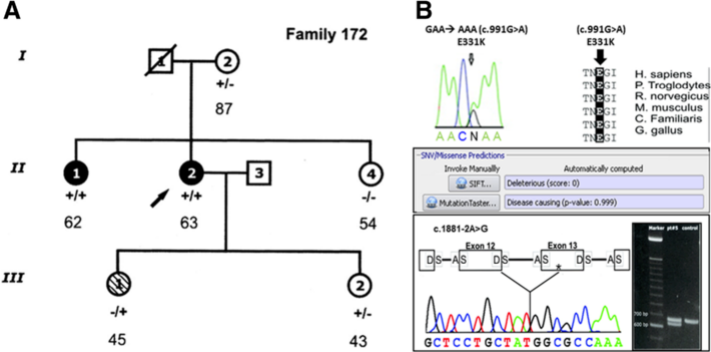
Cascade family segregation and clinical screening is mandatory to define gene mutation association with the disease. **a** Family pedigree of AC. Black, white, and hatched symbols represent clinically affected individuals, unaffected individuals, and individuals of unknown disease status, respectively. Presence (+) or absence (−) of 2 desmoglein-2 (*DSG2*) mutations (c.991 G > A and c.1881 -2A > G) is indicated. Arrow indicate index case. **b** Criteria of testing mutation pathogenicity. Top- Missense mutation c.991 G > A showing aminoacid residue change, absence in large control population (Minor Allele Frequency-MAF), evolutionary conservation of aminoacid residues and *in silico* prediction algorithms. Bottom- splicing site mutation molecular assay showing 2 different trascripts. (modified from Ref.48)

Thus, most of pathogenic mutations address structural proteins that are involved in the organization of the intercalated disc, which has been described as containing a mixed-type junctional structure (the so-called area composita) (Fig. 6) [59]. Comprehensive exonic sequence analysis of the known desmosomal AC-related genes currently identifies approximately 50 % of AC probands [60–63]. The most commonly defective AC gene is PKP2 (10–45 %), followed by DSP (10–15 %), DSG2 (7–10 %) and DSC2, JUP (1–2 %). About 10–25 % of AC patients are compound and heterozygous mutation carriers [60, 65, 66] (Fig. 7). Founder mutations in both desmosomal and non-desmosomal encoding genes have been reported [53, 64, 65].

The inheritance pattern of AC is more complex than previously appreciated, with frequent requirement for more than one ‘hit’ for fully penetrant disease [60, 61, 66, 67]. The low penetrance of AC may be explained by a “recessive-like” inheritance pattern, based on the fact that AC probands often carry homozygous or compound heterozygous variants in the same gene, or digenic/oligogenic variants in a cluster of desmosomal genes.

Nowadays, the pathogenicity of missense and radical (nonsense, frameshifts, splice sites etc) mutations in cardiac diseases and specifically in AC is a matter of debate. Recent studies have shown that stop-coding in disease-causing genes are more pathogenic than missense mutations, since the former are causing alteration of protein length and conformation, leading to haploinsufficiency due to protein instability [67–70]. Entire PKP2 exons or even whole gene deletions have been recently described in AC families with a frequency of approximately 2 % [71–73]. These data are further stressing the question whether haploinsufficiency is enough to determine the disease phenotype.

Genotyping success rate in AC varies according to cohort location and ethnicity, sequencing techniques, selection criteria and the stringency of the standards by which mutations are considered causal. An allelic frequency lower than 0.02–0.05% is considered pathogenic or likely pathogenic. With the routine use of Next Generation Sequencing, the analysis of large panel of genes may lead to the identification of a high number of sequence variants with uncertain clinical significance. Thus, genetic testing and its interpretation should be performed in dedicated AC cardio-genetic centers, with pre- and post-counseling facilities. Given that the prevalence of causal genes and mutations has yet to be determined, a negative genetic test does not exclude a genetic predisposition. The major advantage of a positive genetic test in the affected AC proband consists in the possibility to identify early asymptomatic carriers by cascade genetic screening of family members. Finally, although prenatal diagnosis through amniocentesis is feasible, its application in many countries is subjected to ethical and legal issues; this is even more for pre-implantation diagnosis, which is a long procedure restricted to severe and untreatable diseases.

### Management and treatment

**Fig. 8:**
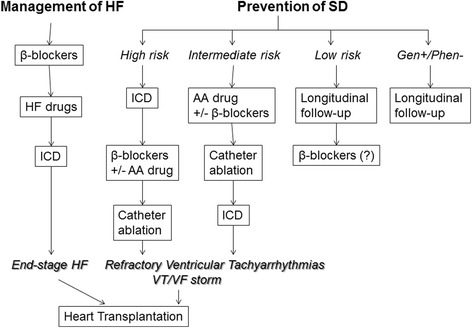
Flow chart for clinical treatment of patients affected by AC (modified from Ref.74). On the left, management of heart failure (HF); on the right, management of ventricular arrhythmias and prevention of sudden death (SD) accordingly to the SD risk category. *Abbreviations. AA: antiarrhythmic ICD : Implantable cardioverter defibrillator; VT : ventricular tachycardia; VF : ventricular fibrillation; Gen+/Phen- : Genotype positive/phenotype negative*

The most important goals of clinical management of AC patients comprise: 1) reduction of mortality, either by arrhythmic SD or death due to heart failure; 2) prevention of disease progression leading to RV, LV or biventricular dysfunction and heart failure; 3) attenuation of symptoms and improvement of quality of life by decreasing or suppressing palpitations, VT recurrences or implantable cardioverter defibrillator (ICD) discharges (either appropriate or inappropriate); and 4) reducing heart failure symptoms and increasing exercise capacity [74]. The AC management options regard life-style modifications, pharmacologic treatment, catheter ablation, ICD implantation, and exceptionally heart transplantation [75, 76]. Figure 8 summarizes the flow chart for clinical treatment of AC.

Before any therapy is undertaken, life-style modification should be pursued. Sport activity is associated with an increased risk of SD, thus supporting the concept that avoiding effort is “per se” life-saving [1, 6, 7]. Recently, it has been demonstrated that endurance sports and frequent exercise increase age-related penetrance, risk of VTs, and occurrence of heart failure in AC desmosomal gene carriers [77, 78]. Pregnancy is generally well tolerated but a pre-conception evaluation is mandatory for individualized arrhythmic risk stratification and prescription of the best antiarrhythmic therapy [79]. β--blockers treatment is better since no teratogen effects are known but they may be associated with intrauterine growth retardation and neonatal bradycardia or hypoglycemia [75].

Different *antiarrhythmic drugs* have been employed, such as sodium blockers, β-blockers, sotalol, amiodarone, verapamil alone or combinations. Wichter et al. [75] reported various efficacy rates by demonstrating that sotalol in a dosage of 320-480 mg/day is the most powerful anti arrhythmic drug for inducible and non-inducible VT in AC patients; amiodarone is less effective and has a high risk of extracardiac side effects during long-term follow-up; β-blockers are effective only in non-inducible patients. Co-administration of more than one drug should be avoided. However, contradictory data regarding the effectiveness of empiric anti-arrhythmic drugs have been published, showing either a higher efficacy of amiodarone or inefficacy of anti-arrhythmic drugs against ICD intervention and SD [76, 80]. Differences in the study populations, drug doses, therapeutic approach (empiric vs. guided by electrophysiologic study/Holter) and follow-up duration may explain the divergence [80].

*Catheter ablation* of the re-entry circuit is a non pharmacological therapeutic option for AC patients who have VT. In fact, VT is the result of a scar-related macro-reentry circuit due to RV fibro-fatty replacement, which is suitable for mapping and interruption by catheter ablation [81–84]. Catheter ablation may be guided by either conventional electrophysiology or substrate-based mapping. Linear ablation lesions connecting or encircling ventricular scar areas obtain the isolation of the re-entry circuit. In the presence of a large RV scar burden and/or in patients with VT recurrence, combined endo- and epi- substrate-based VT ablation, incorporating scar dechanneling technique, would increase the short- and long-term success rate. However, the epicardial approach has a significant procedural complication rate (up to 8 %) and should be always performed in high volume referral centers [84].

*ICD* therapy is the first line approach for the highest-risk patients, whose natural history is typically characterized by the risk of SD [27, 80, 85–87]. Data obtained from either primary or secondary prevention studies indicate that ICD therapy improves long-term outcome of selected high-risk AC patients, with an estimated mortality reduction ranging from 20 to 30 %. Overall, 48–78 % of patients received appropriate ICD interventions during the mean follow-up period of 2–7 years after implantation [27, 87]. The published studies on ICD therapy in AC patients have provided also valuable information about the risk predictors for VF or VT triggering appropriate ICD discharges during follow-up. A stronger predictor of a life-saving ICD intervention was aborted SD due to VT/VF and syncope. Other risk factors associated with an increased risk of appropriate ICD interventions included hemodynamically well tolerated VT, either sustained or non-sustained, severe RV and/or LV dysfunction, young age, proband status, frequent polymorphic PVCs (≥1000/24 h), and inducibility at programmed ventricular stimulation (PVS). The presence of multiple risk factors increases the likelihood of appropriate ICD therapy. Most importantly, asymptomatic probands and relatives without relevant risk factors as well as healthy gene carriers showed a low rate of arrhythmic events over a long-term follow-up, regardless of family history of SD. Despite well-known ICD benefit on survival, disadvantages are related to the lead and device-related complications as well as the inappropriate ICD intervention, which occurs in 10–25 % of AC patients and is usually caused by sinus tachycardia or atrial tachyarrhythmia [27, 87]. The inappropriate interventions are painful and, especially in young patients, may have a severe psychological impact hindering the compliance to ICD therapy. When the incidence of inappropriate ICD discharges is too high, the patients can take advantage by appropriate ICD reprogramming and/or co-administration of β-blockers therapy.

#### Cardiac transplantation

AC patients with severe, refractory biventricular heart failure or unmanageable VTs may become candidate to heart transplantation. The most common indication for cardiac transplantation is heart failure, and, in less than one-third of patients, unbearable ventricular arrhythmias [3, 88].

### Risk stratification

**Fig. 9:**
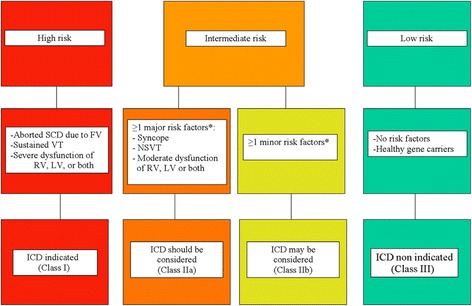
Flow chart of risk stratification and indications to ICD in AC. The estimated risk of major arrhythmic events in the high-risk category is >10 %/year, in the intermediate-risk category ranges from 1 to 10 %/year, and in the low-risk category is <1 %/year. The high risk category includes patients who experienced cardiac arrest due to VF or sustained VT and most benefit from ICD (estimated annual event rate >10 %/year). The low risk category comprises probands and relatives without risk factors as well as healthy gene carriers (estimated annual event rate <1 %/year), who do not require any treatment. The intermediate risk category includes AC patients with ≥1 risk factors, except those mentioned in the high risk category (estimated annual event rate between 1 and 10 %/year). The decision to implant an ICD in these patients should be made on individual basis (from Ref.87, with permission). *Abbreviations. SCD: sudden cardiac death; VF: ventricular fibrillation; VT: ventricular tachycardia; RV: right ventricle; LV: left ventricle*

Arrhythmic risk stratification relies on phenotypic predictors, such as previous cardiac arrest due to VF, sustained VT, unexplained syncope, severe RV or LV dilatation/dysfunction, compound and digenic heterozygosity of desmosomal gene mutations, low QRS amplitude, QRS fragmentation, male gender, young age at time of diagnosis, proband status, inducibility at PVS, burden of electroanatomic scar and scar-related fractioned electrograms, extent of T wave inversion across precordial and inferior leads on ECG. In a recent document on risk stratification and treatment of AC [87], indications for ICD implantation were determined by consensus taking into account the statistical risk, the general health, the socioeconomic factors, the psychological impact and the adverse effects of the device. The flow chart is represented in Fig. 9.

Noteworthy, these recommendations apply to the classical RV AC variant and prognostic data are not yet available in LV AC, which is increasingly detected by contrast-enhanced CMR.

### Pathogenesis of AC

Transgenic animal models that mimic the human AC phenotype (mice and zebrafish) and induced pluripotent stem cells (iPSCs) from affected patients are useful tools to explore how the mechanical and/or functional disruption of cell junctions by mutant desmosomal proteins leads to cardiomyocyte death and subsequent repair with fibrous and fatty tissue.

#### Abnormal cell-cell adhesion

Desmosomes mediate cell–cell adhesion through three families of proteins, i.e. the armadillo proteins (junction plakoglobin and plakophilins), cadherins (desmocollins and desmogleins), and plakins (desmoplakin). Even before the discovery of desmosomal genes in AC, electron microscopy studies demonstrating intercalated disc remodelling first raised the hypothesis of an abnormal cell-cell adhesion in disease pathogenesis [89, 90]. More recently, Sato et al., using monolayers of neonatal rat ventricular myocytes in which PKP2 was silenced and subjected to a defined mechanical intervention, demonstrated a reduced cell-cell adhesion [91]. However, when expressing mutant forms of either PKP2 or JUP, cells exhibited abnormal signaling in response to mechanical stress, but showed a preserved intercellular adhesion, thus questioning a primary role of cell-cell adhesion in AC pathogenesis [92]. At the same time, Asimaki et al. [93] demonstrated that a reduced junctional signal for JUP appears to be a hallmark of the disease in myocardial samples from AC patients, pointing to its possible role in intracellular signaling rather than adhesion, as suggested by other groups [94, 95].

#### Abnormal intercellular junction proteins and intracellular signaling

The role of intracellular signaling, with the Wnt signaling pathway suppression leading to adipogenesis, as a consequence of the abnormal distribution of intercalated disc proteins, was first demonstrated by Marian group [94]. In a Dsp-deficient mouse model, the authors showed suppression of the canonical Wnt/β-catenin/Tcf/Lef pathway, a known regulator of adipogenesis, fibrogenesis and apoptosis. Knockdown of DSP in HL-1 cells causes the translocation of JUP into the nucleus, where it interferes with β-catenin/TCF transcriptional activity, leading to an adipogenic switch. Thereafter, by using genetic fate-mapping methods, the same group demonstrated that most of the adipocytes in AC originate from cardiac progenitors cells of the embryonic second heart field [96]. Furthermore, in mice overexpressing cardiac truncated JUP, suppression of the canonical Wnt signaling pathway and induction of proadipogenic genes expression due to nuclear translocation of JUP led to adipogenesis in c-kit + cardiac progenitor cells [97].

Recently, also the Hippo/YAP signaling pathway has been associated to AC pathogenesis. In the nucleus, YAP interacts with β-catenin to drive Wnt-related gene expression. In AC patients myocardial samples, mouse model and pkp2 knockdown HL-1 myocytes, Chen et al. [98] demonstrated aberrant activation of the Hippo kinase cascade resulting into phosphorylation and cytoplasmic retention of YAP; this causes β-catenin and JUP cytoplasmic sequestration, with further suppression of the canonical Wnt signaling leading to enhanced myocyte death and fibro-adipogenesis.

Cellular reprogramming of patient-derived somatic cells (i.e. dermal fibroblasts) into iPSCs has enabled the generation of human cardiomyocytes for *in vivo* modeling. Among the others, Kim et al. [99], by studying iPSCs-derived cardiomyocytes from AC patients with PKP2 mutations, demonstrated that the abnormal JUP nuclear translocation and decreased β-catenin activity is insufficient to reproduce the pathologic phenotype in standard conditions. Only the induction of an adult-like metabolism in a lipogenic milieu co-activated PPAR-γ pathway with lipogenesis, apoptosis and calcium-handling deficit [99].

Noteworthy, all transgenic experimental animal models and iPSC-derived cardiomyocytes demonstrated only abnormal “lipogenesis”, but not adipocyte formation/transdifferentiation. Thus, cells other than cardiomyocytes must be involved in the abnormal adipogenesis and fibrosis which is an essential feature of AC phenotype [100]. A role of cardiac mesenchymal stromal cells as a source of adipocytes in AC has been recently suggested [101].

#### Gap junction and ion channel remodelling

Desmosomes, gap junctions and sodium channels act as a functional triad in which changes in the composition of one constituent can affect the function and integrity of the others [4]. Diminished expression of connexin-43 at intercellular junction was demonstrated in most of AC cases, suggesting that impaired mechanical coupling might also account for abnormal electrical coupling through gap-junction remodelling. Moreover, cardiac sodium current was found to be reduced in experimental models of AC [28, 29, 102–104]. These findings led to hypothesize that life-threatening ventricular arrhythmias could occur in AC patients, even preceding the structural abnormalities (pre-phenotypic stage) due to electrical uncoupling and reduced sodium current. However, this hypothesis remains to be proven in human AC patients, where only a reduced immunoreactive signal at intercalated disc for the major protein subunit of the sodium channel Nav1.5 has been demonstrated [103].

#### From experimental models to target therapy

In a transgenic zebrafish model of AC with cardiac specific expression of human JUP deletion, high-throughput drug screening identified SB216763, a compound able to prevent heart failure and normalize survival [104]. Its efficacy has been further supported by experiments in neonatal rat ventricular myocytes expressing the same JUP mutation and in cardiac myocytes derived from iPSCs of two PKP2 mutation carriers. Treatment with SB216763 restored the subcellular distribution of JUP, connexin-43 and Nav1.5 and of SAP97, a protein known to mediate the forward trafficking of Nav1.5 and Kir2.1. Noteworthy, SB216763 was already known as an activator of the canonical Wnt signaling pathway and this study confirms an abnormal protein trafficking at the intercalated discs, rather than altered protein production in AC. These data, together with those by Lombardi et al. [96] and by Kim et al. [99], who were able to prevent cell degenerative changes using another Wnt activator, i.e. 6-bromoindirubin-3’-oxime, open the door to the identification of a curative therapy in AC, by targeting a final common pathway of disease pathogenesis.

## Conclusions

Many advances have been made in the clinical diagnosis and management of AC in the last 10 years. While the classical RV variant of AC is nowadays easily recognized, thanks to updated diagnostic criteria, LV variants are increasingly identified, thus supporting the use of the broader term AC. Abnormal trafficking of intercellular proteins and Wnt/beta catenin and Hippo signaling pathways have been implicated in the pathogenesis of this inherited rare cardiomyopathy, which is mostly due mutations of genes encoding for intercalated disc proteins. Ongoing research is focused on the understanding of disease pathobiology in search of a curative therapy able to stop disease progression.

## Abbreviations

AC: Arrhythmogenic Cardiomyopathy
ARVC/D: Arrhythmogenic RV Cardiomyopathy/Dysplasia
BSA: body surface area
CMR: cardiac magnetic resonance
CTNNA3: αT-catenin
DES: desmin
DSC2: desmocollin-2
DSG2: desmoglein-2
DSP: desmoplakin
ECG: electrocardiography
EMB: endomyocardial biopsy
EPS: electrophysiologic study
iPSCs: induced pluripotent stem cells
JUP: plakoglobin
LBBB: left bundle branch block
LE: late enhancement
LMNA: Lamin A/C
LV: left ventricular
PKP2: plakophilin-2
PLAX: parasternal long-axis view
PLN: phospholamban
PSAX: parasternal short-axis view
PVC: premature ventricular complex
PVS: programmed ventricular stimulation
RBBB: right bundle-branch block
RV: right ventricular
RVOT: RV outflow tract
RYR2: ryanodine receptor 2
SAECG: signal averaged ECG
SCD: sudden cardiac death
SD: sudden death
TGFB3: transforming growth factor beta 3
TMEM43: transmembrane protein 43
TTN: titin
VF: ventricular fibrillation
VT: ventricular tachycardia
WMA: wall motion abnormalities
